# Cervical Discogenic Pain Treatment with Percutaneous Jellified Ethanol: Preliminary Experience

**DOI:** 10.1155/2019/2193436

**Published:** 2019-03-13

**Authors:** Haytham Eloqayli

**Affiliations:** ^1^Department of Neurosurgery, Faculty of Medicine, Jordan University of Science and Technology (JUST), Irbid 22110, Jordan; ^2^Emirates Specialty Hospital, Dubai Healthcare City, P.O. Box 66566, Dubai, UAE

## Abstract

**Background:**

Percutaneous DiscoGel® (Gelscom SAS, France), introduced in 2007 as a promising new minimal invasive technique, showed efficacy and safety in lumbar spine surgery, with limited use and scientific reports with regard to the cervical spine. Since the first publication of its use on the cervical spine (2010), less than 100 cases have been published. We introduce an initial experience with this relatively new procedure. We hypothesized that percutaneous DiscoGel® is a safe and effective option for chronic neck pain of cervical discogenic origin.

**Method:**

This was a clinical study on 10 patients with chronic discogenic pain operated on for 18 cervical discs with percutaneous DiscoGel®. Inclusion criteria were patients with chronic axial or referred neck pain with MRI showing a cervical disc that is consistent with patient symptoms and failed conservative treatment. Exclusion criteria were clinical myelopathy, motor deficit, severe stenosis or reduced disc height by more than 50%, or previous cervical spine surgery.

**Results:**

A total of 10 cases consisting of 6 females and 4 males underwent treatment with percutaneous DiscoGel® for 18 cervical discs. C5/C6 was the most affected level. The mean preoperative VAS score was 8; the postoperative VAS scores at 6 weeks and 3 months were 2.2 and 2.9, respectively. There were no postoperative complications or neurological deficits.

**Conclusion:**

The present study has the limitation of the small number of cases; however, with the limited number of studies and less than 100 published cases in the literature, this initial work shows that cervical percutaneous DiscoGel® is an effective minimally invasive bridging option between conservative and open surgical treatment for cervical discogenic pain, with a high success rate. The differentiation of pain types (nociceptive, referred, radicular, and trapezius myalgia) that can coexist is crucial for procedure selection and improving treatment outcome.

## 1. Introduction

Cervical discogenic pain is a heterogeneous complex pain syndrome that originates from degenerated intervertebral discs (IVD) [1]; however, there are two findings raised in the literature and clinical practice that need elucidation. The first is the lack of specific correlation between image findings and pain with frequent occurrence of asymptomatic disc degeneration, while the second is that different pain types with different mechanisms can coexist even in the same patient [2]. In clinical practice, the aim of surgery is pain relief and return of patient function rather than correction of image changes; therefore, in patients failing conservative treatment, minimally invasive techniques have been introduced as a bridge between conservative and classical surgical treatment with the aim of decreasing risk and reducing hospital stay [3]. Chemonucleolysis was one of the effective disc percutaneous treatments that aimed to partially dissolve nucleus pulposus, decrease intradiscal pressure, and induce shrinkage of the disc. Chymopapain: this classical example was discontinued due to anaphylactic reactions [4]. Pure ethanol was used as an alternative chemonucleolytic agent; however, its effect was short-lived with rapid leakage [5].

DiscoGel® (Gelscom SAS, France) is an intradiscal medical device composed of jellified ethanol associated with tungsten in suspension for percutaneous nucleolysis. It was introduced in 2007 for treatment of pain from a lumbar disc that failed conservative treatment with absence of neurological deficit [6]. Since the first published report in 2010 reporting the application of DiscoGel® on the cervical spine, less than 100 cases have been reported: 57 cases with 89% success [7], 9 cases with a success rate of 83% [8], and 33 patients with an 82% success rate [4]. Moreover, most of the knowledge on cervical spine pain generation is adapted from medical literature on the lumbar spine, ignoring the innate microanatomical and embryological differences in annulus fibrosis and supporting muscles, which might affect pain generation and appropriate treatment techniques [9].

In the presence of the limited number of studies with a limited number of cases addressing use of DiscoGel® in a cervical disc, we report our preliminary experience. Moreover, due to the confusion with pain types, patterns, and generators from the cervical spine, we added a short summary in Discussion, addressing chronic cervical-related pain. The number of cases is small; however, the present study is preliminary research for a new technique with less than 100 cases published on the cervical spine.

## 2. Methods

This was a clinical case series study. Participants were patients who were 18-60 years of age with chronic axial or referred neck pain who failed conservative treatment with MRI showing a cervical disc that was consistent with patient symptoms. Exclusion criteria were clinical myelopathy, motor deficit, radiological severe stenosis or reduced disc height by more than 50%, or previous cervical spine surgery.

Each patient was evaluated at timed intervals: before the procedure (baseline) and at 1 day, 2 weeks, 6 weeks, and 3 months using the VAS pain score. Baseline, 6-week, and 3-month VAS score changes are presented. Approval of the ethics committee was obtained. Informed consent from the patients was obtained.

## 3. Techniques

Procedures were performed as described by Thernon et al., 2010, under conscious sedation, aseptic surgical conditions, and fluoroscopy guidance. We gave an intravenous injection of an antibiotic 45 minutes prior to the surgical procedure. Patients were placed in the supine position, with the neck slightly hyperextended with a folded towel between the shoulder blades. A right anterolateral approach was taken in all cases to avoid any harm to the esophagus. We marked the skin puncture point with fluoroscopic guidance. We felt the carotid pulsation laterally under the sternocleidomastoid and the trachea medially and then applied firm pressure with the second and third fingers to feel the cervical spine. We then inserted spinal needles (20-gauge, 10 cm) through the space created by placing two fingers and adjusted the needle trajectory to the target disc to puncture the annulus between the uncovertebral joint and midline sloping under the lip of the upper cervical vertebrae. We confirmed appropriate position and depth with AP and lateral x-ray views. A diskography was performed with adjustment of a beveled needle tip to allow better spread of contrast. DiscoGel® was injected slowly over 5 minutes in a dose of 0.2–0.3 mL per cervical disc, and the needle was then left in place for 90 seconds before being removed. Patients were monitored for one hour after the procedure in the postanesthetic care unit and then transferred to the general ward. The patients were discharged on anti-inflammatories, mild muscle relaxants, analgesic drugs, and antibiotic prophylaxis.

## 4. Results

Patients' age, gender, disc levels, and VAS pain score are summarized in Table 1. A total of 10 cases consisting of 6 females and 4 males underwent treatment with percutaneous DiscoGel® for 18 cervical discs. The mean age of the patients was 46.2 (32-59) years. All patients presented with axial neck pain with referred pain components. The mean preoperative VAS score was 8.1, whereas the 6-week and 3-month posttreatment mean VAS scores were 2.2 and 2.7, respectively. The most common degenerated disc was C5/C6. No patient had complications or neurological deficit postoperatively. All patients were discharged the same day.

## 5. Discussion

Pain from a degenerated spine is complex and heterogenous with lack of specific correlation between image disc changes and pain. Clinical correlation to image changes is mandatory to avoid overdoing unnecessary surgery. A cervical spine disc causes multiple pain types and patterns with different mechanisms that can coexist; however, a major part of the knowledge on a cervical spine disc and pain is adapted from research on the lumbar spine, thereby ignoring some unique features of the cervical spine, such as the crescent shape anteriorly thick posteriorly thin cervical disc annulus [10], and different embryology and innervation of cervical muscles that are arranged in three layers: somite epimere dorsal rami innervated deep intrinsic cervical muscles, somite hypomere ventral rami innervated extrinsic spine muscles (i.e., levator scapulae, rhomboids), and nonsomite lateral mesenchymal plate originating trapezius muscle [11]. Consequently, understanding the pathogenesis of cervical pain generation and types can help in the selection of minimally invasive techniques that decrease pain and improve function, thereby decreasing the need for open surgery. Application of percutaneous DiscoGel® is a promising procedure that showed efficacy and safety in the lumbar spine, with limited use and scientific reports for cervical discogenic pain.

Controversy still exists regarding mechanisms of discogenic pain generation, with possible contribution from neovascularization, inflammation, mechanical microinstability, compression, ingrowth of free nerve endings to the depth of the annulus, increased intradiscal pressure, imbalance between matrix metalloproteinases and tissue inhibitors of metalloproteinases, stretching, and tears in annulus fibrosis [12]. Therefore, pain types, mechanisms, and generators are elucidated in the next section, followed by discussion on cervical percutaneous DiscoGel®.

### 5.1. Cervical Pain Types

Chronic cervical-related pain can be subdivided into the following categories that can coexist and possibly influence each other:

1. Radicular pain and radiculopathy: this is correlated to disc herniation with irritation (inflammation) and/or compression of the nerve root and dorsal root ganglia (DRG). This is a neuropathic type of pain that can follow a dermatomal distribution or be described deep inside the limb with a lancing, searing, or internal pressure nature
2. Axial deep pain: this is a nociceptive pain type that can originate from any bony or soft tissue structure in the neck but commonly from degenerated a disc and facet joint. This is an inflammatory pain type where excessive stimuli activate nociceptors (free nerve endings), causing both acute and chronic pain mediated via A delta and C fibers with both central and peripheral sensitization complicating the clinical presentation
3. Referred pain: this is a component of nociceptive pain perceived in a remote region that shares common segmental innervation or 2^nd^ order neuron. This pain referral commonly is cutaneous or muscular but it can be perceived in all other structures depending on the embryological innervation. Referred pain should be differentiated from neuropathic radicular pain where nerve root injury is the primary cause. Treatment of referred pain is via identification and management of the nociceptive generator
4. Trapezius myalgia: physical and psychological factors predispose to chronic trapezius myalgia. This is a separate entity of pain type with debate on the mechanism and pain pathway [13]. The trapezius muscle embryological origin is from the nonsomite lateral mesenchymal plate with motor innervation from the XI cranial nerve and sensory innervation from the ventral rami of C3 and C4. Debate exists on the contribution of the cervical plexus to motor innervation of the trapezius. Myofascial pain perceived on the medial side of the scapula should not be confused with trapezius myalgia. The first is referred pain in the hypomere ventrally innervated muscles (i.e., rhomboids, levator scapulae) that share common innervation with the annulus [14], whereas the latter is pain in the upper trapezius, with debate on mechanism and neural pathway

### 5.2. Percutaneous Discectomy with Jellified Alcohol

Theron et al., 2007, introduced DiscoGel® [6] as an alternative to surgical treatment for lumbar disc presenting with chronic pain and failed conservative treatment without neurological deficit. The average success rate was 89% for 276 lumbar disc cases, with no complications.

Stagni et al., 2012 [15], showed that DiscoGel® is safe and easy to handle with no complications and good improvement in 24 out of 32 treated lumbar discs. De Zesa et al. [16] reported a 75% success rate in 79 lumbar discs, with no sensory or motor impairments. A correlation between MRI Modic type I signal and treatment efficacy with DiscoGel® has been shown in the lumbar disc with a lower success rate in 35 lumbar cases compared to other reports [17]. The higher treatment response rate by Theron, 2007, was attributed to local corticoid injection and shorter duration between pain onset and application of percutaneous nucleolysis. The use of DiscoGel® for lumbar radicular pain was safe and effective [18]. Bellini et al., 2015 [8], reported improvement in 85% of 73 lumbar disc patients and 83% of nine cases of cervical discs with percutaneous DiscoGel®; they were followed up for 3 months.

The cervical disc received less attention with tendency for applying the knowledge gained from treating the lumbar disc and pain to the cervical spine. Theron et al., 2010, published the outcome of cervical percutaneous DiscoGel® application for 57 patients, showing an 89.5% success rate in the form of resolution or significant pain reduction after 6 weeks of follow-up; however, a limited number of studies and cases addressed the application of DiscoGel® to the cervical spine: 57 cases with 89% success [7], 9 cases with a success rate of 83% [8], and 33 patients with an 82% success rate [4]. The current study reports a similar success rate with no reported complications. Moreover, C5/C6 are the most affected discs in the current study, which fits with previous reports [19].

Steroid injection, duration of symptoms, and containing of the disc have been reported as factors that can influence treatment response. We add that type of pain (nociceptive, neuropathic, referred, or myofascial) and type of changes in the treated disc (degenerated versus herniation) need to be considered in cervical pain diagnosis and treatment selection. Degenerated discs cause mainly discogenic nociceptive axial pain with referred components through decreased disc pressure, inflammation, neovascularization, and ingrowth of free nerve endings, whereas herniated soft discs cause mainly neuropathic radicular pain from compression and inflammation of the nerve root and DRG. Contained soft discs produce pain via increased intradiscal pressure, stretching of the annulus laminae, and inflammation to the nerve root and DRG from leaked nucleus pulposus fluids [20]. Consequently, the percutaneous procedure should address the pain mechanism. In the present study, discogenic pain from cervical degenerated discs with axial nociceptive and referred pain was selected with possible mechanisms for percutaneous jellified alcohol of acting as anti-inflammatory, chemical damage to the neovascularization granulation vessels and ingrowing free nerve endings. The cervical spine is less weight-bearing compared to the lumbar spine, with the main pain generator (thicker anterior region of the annulus) being directly addressed. Therefore, with proper patient selection, the success rate for cervical discogenic pain might be better than for the more weight-bearing lumbar pain where multiple factors can add to the complexity.

This minimally invasive cervical percutaneous discectomy has the advantages of shorter hospitalization, less tissue disruption, less risk, less or no postoperative adhesions, no skin scar, no risk of instability, and shorter recovery period. Therefore, in patients failing conservative treatment with absent neurological deficit, this procedure can be an option before going for open surgery.

Our study has the limitation of small sample numbers and short follow-up period; however, in view of limited experience and reports in the literature for the use of this technique in cervical discs with less than 100 cases reported, we hope this report can add to the limited knowledge about this issue. Disc and endplate image grading (i.e., Modic, Pfirrmann) is not mentioned in the present study as the sample size is small, which precludes drawing correlations; however, it should be mentioned here that correlation of endplate and disc changes with axial pain is controversial even in larger studies and systemic reviews [21]. Moreover, future research should address the clinical pain types from cervical spine degeneration, which may mandate a clinical and treatment-based classification rather than only image-based classification that might lead to disc disease overdiagnosis [22].

## 6. Conclusion

This initial pilot study indicates that percutaneous jellified alcohol DiscoGel® chemonucleolysis is a safe and efficient procedure for selected patients with chronic discogenic axial nociceptive and referred pain from a cervical disc. For patients with failed conservative treatment, in the absence of neurological deficit or myelopathy, this procedure can be a bridging option between conservative medical treatments and surgery.

## Figures and Tables

**Table 1:** Demographic, disc level, and visual analogue scale (VAS) score data of the patients who were treated with percutaneous DiscoGel®.

	Age	Gender	Disc level	Pretreatment VAS	6-week posttreatment VAS	3-month posttreatment VAS
	54	F	C4/C5, C5/C7	9.1	2.6	2.8
	51	F	C4/C5, C5/C6	7.7	2.2	2.9
	50	M	C4/C5, C5/C6	9	2.5	3.2
	41	F	C4/C5, C5/C6	8.8	1.4	2.3
	48	F	C5/C6	8.2	2.3	3.1
	32	F	C4/C5, C5/C6	7.8	3.9	4.4
	50	M	C5/C6, C6/C7	8.6	1.5	1.8
	43	F	C6/C7	6.7	1.4	2.3
	59	M	C4/C5, C5/C6	6.5	2.5	3
	51	M	C6/C7	7.3	1.5	3
Mean	47.9			7.97	2.1777778	2.8777778
Standard deviation	7.5490985			0.931009	0.8288211	0.7361688

## Data Availability

The data used to support the findings of this study are available from the author upon request.
